# Supplementary material for: A patient-based iPSC-derived hepatocyte model of alcohol-associated cirrhosis reveals bioenergetic insights into disease pathogenesis
Source: Nat Commun. 2024 May 1;15:2869. doi: 10.1038/s41467-024-47085-y (PMC11063145; doi:10.1038/s41467-024-47085-y)
Supplement: Supplementary file 1 — Supplementary Information [file 41467_2024_47085_MOESM1_ESM.pdf]

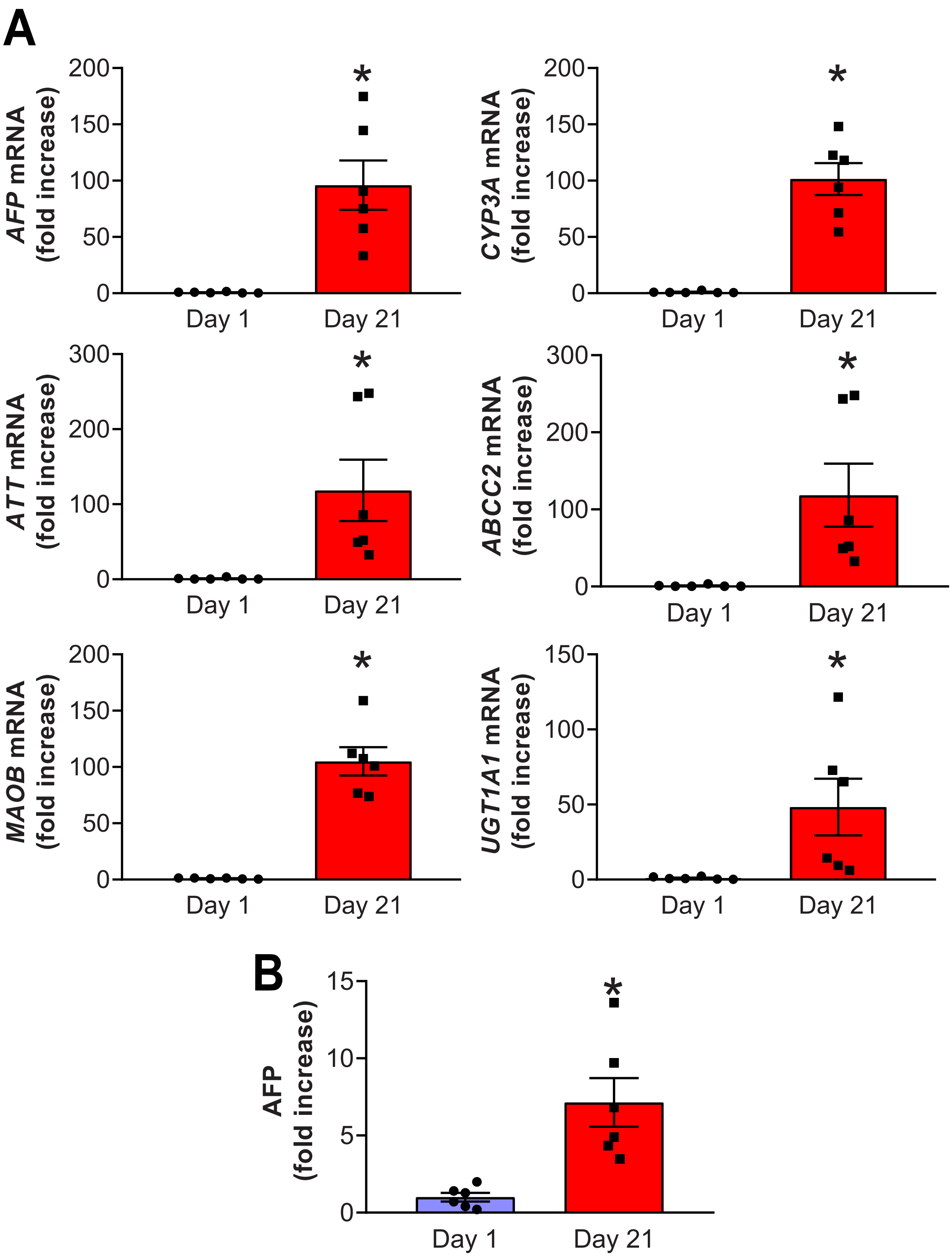
A
AFP mRNA (fold increase)
CYP3A mRNA (fold increase)
ATT mRNA (fold increase)
ABCC2 mRNA (fold increase)
MAOB mRNA (fold increase)
UGT1A1 mRNA (fold increase)
Day 1
Day 21
*
B
AFP (fold increase)
Day 1
Day 21
*

FigS1

Supplementary Fig 1. Characterization of patient-derived iHLCs with maturation markers at Day 1 and Day 21 of hepatocyte differentiation A. Real-time PCR demonstrated significantly increased expression of transcripts of AFP (95.21 ± 21.93), CYP3A (100.4 ± 14.21), ATT (117.5 ± 40.81), ABCC2 (117.5 ± 40.81), MAOB (104.0 ± 12.60) and UGT1A1 (47.25 ± 18.88) from Day 1 to Day 21 from iPSC-to-iHLCs differentiation. Fold changes are provided for each transcripts with SEM and n = 6/group B. Measurement of AFP by ELISA from cell lysate at day1 and day 21. The data are expressed as fold change with n = 6/group.

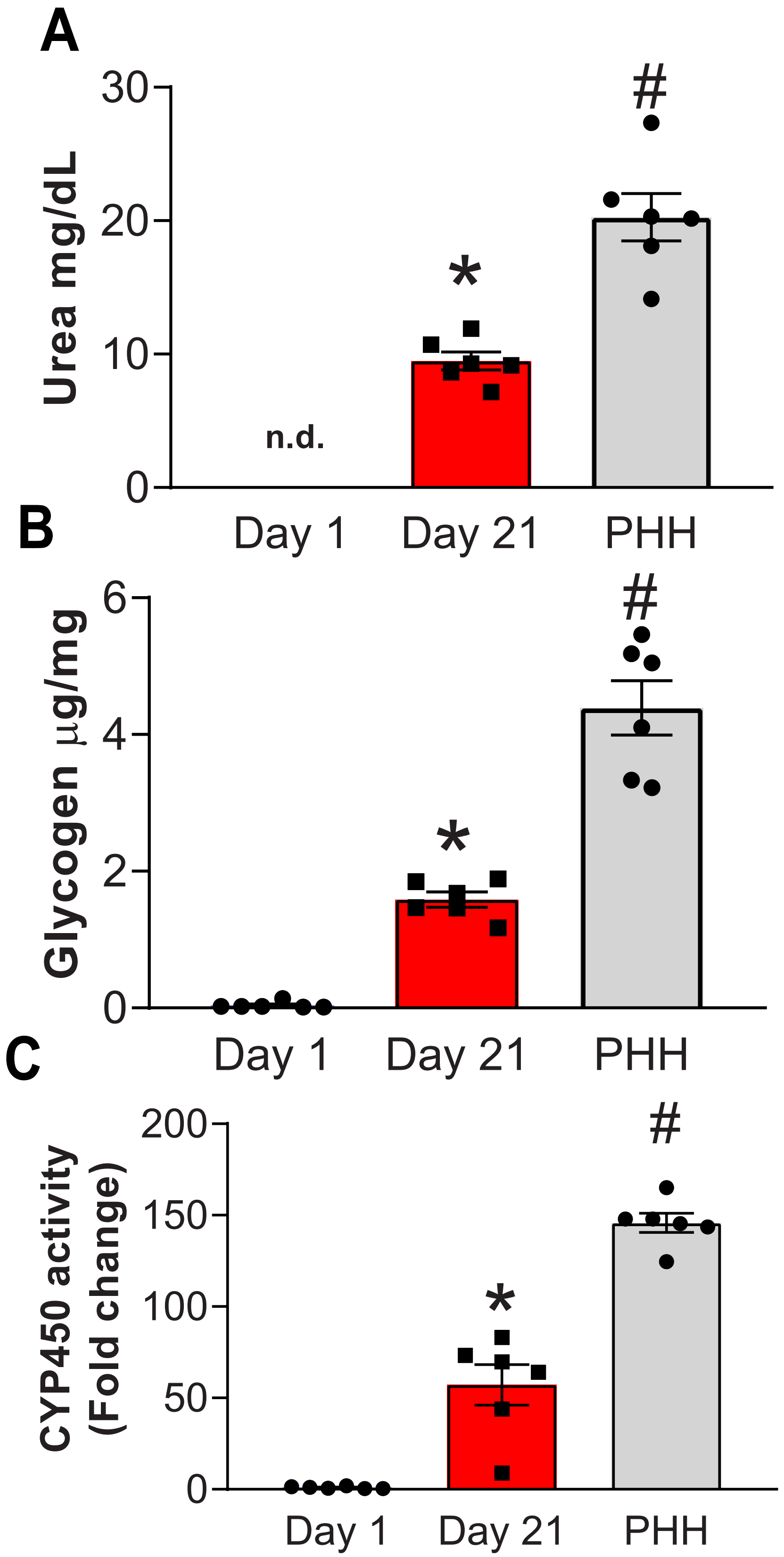
A
Urea mg/dL
30
20
10
0
n.d.
*
#
Day 1
Day 21
PHH
B
Glycogen μg/mg
6
4
2
0
*
#
Day 1
Day 21
PHH
C
CYP450 activity
(Fold change)
200
150
100
50
0
*
#
Day 1
Day 21
PHH

FigS2

Supplementary Fig 2. Characterization of patient-derived iHLCs and primary human hepatocytes (PHH) for functional parameters of hepatocytes. A. Measurement of urea secretion from cultural supernatant B. Gycogen storage activity from cell lysates at day 1, 21 and PHH. C. CYP450 enzyme activity were determined in Day 1, 21 and PHH. $n = 6$/group.*$P < 0.001$ compared to Day 1 of iHLCs group, # $P < 0.001$ compared to Day 21 of iHLCs .

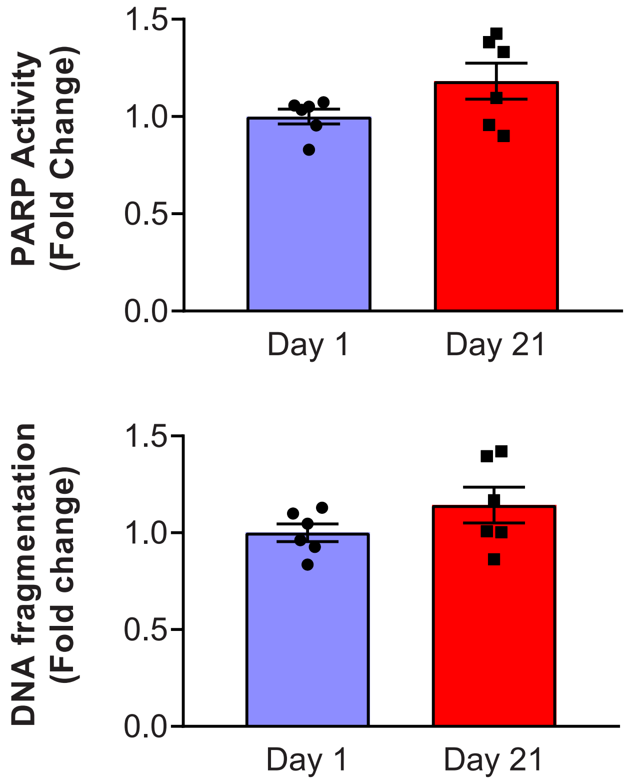

Supplementary Fig 3. Cellular stress at Day 1 and Day 21 via PARP1 activity and DNA fragmentation. There was no statistical difference between H iHLCs and AC iHLCs in response to both cellular stress markers of PARP1 activity and DNA fragmentation, n = 6/ groups.

FigS3

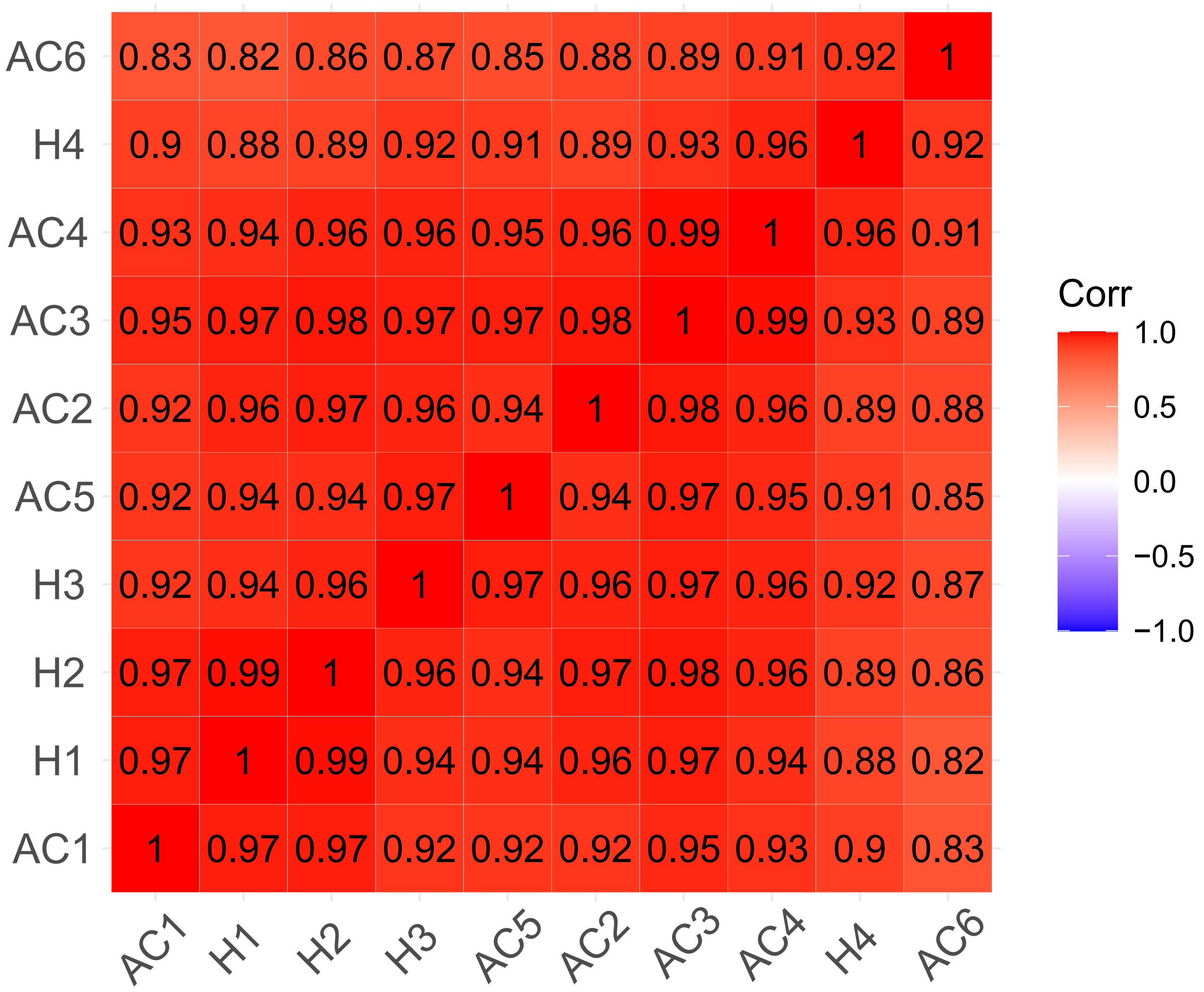

Supplementary Fig 4. High degrees of transcriptomic similarity between iHLCs derived from healthy controls and AC patients, and across batches of iHLCs. Correlation plots and correlations between all pairs of iHLC trancriptomes.

FigS4

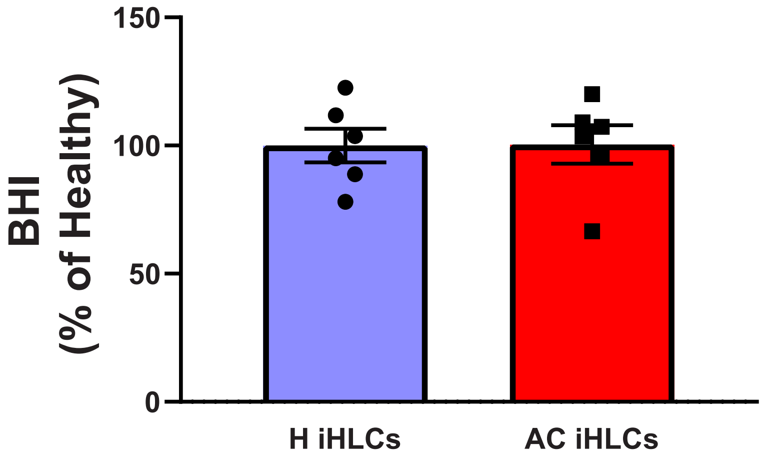

FigS5

Supplementary Fig 5. BHI index computed from OCR data. We observed no significant differences in BHI between H iHLCs and AC iHLCs. n = 6/groups.

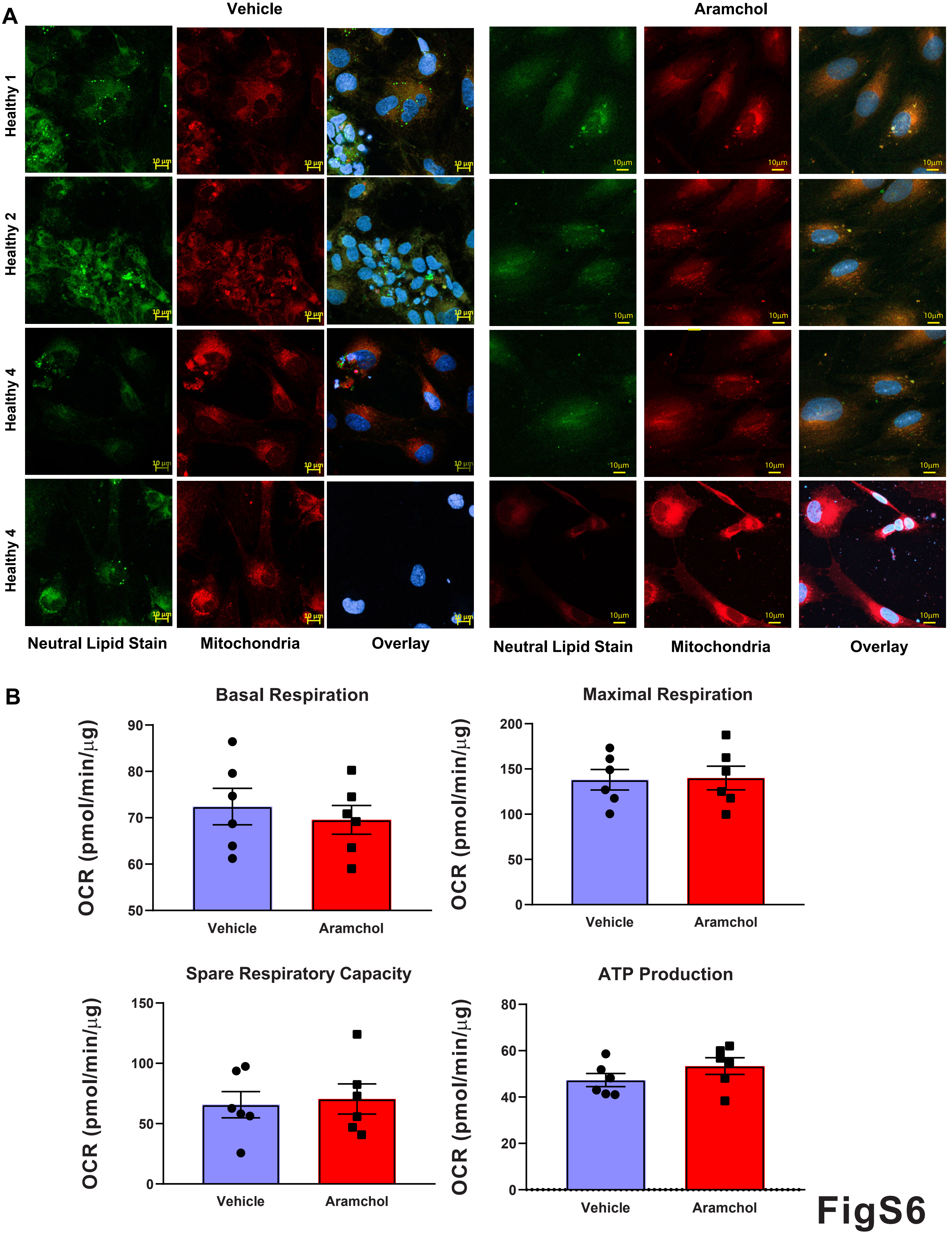
A
Vehicle
Aramchol
Healthy 1
Healthy 2
Healthy 4
Healthy 4
Neutral Lipid Stain
Mitochondria
Overlay
Neutral Lipid Stain
Mitochondria
Overlay
B
Basal Respiration
OCR (pmol/min/µg)
50
60
70
80
90
Vehicle
Aramchol
Maximal Respiration
OCR (pmol/min/µg)
0
50
100
150
200
Vehicle
Aramchol
Spare Respiratory Capacity
OCR (pmol/min/µg)
0
50
100
150
Vehicle
Aramchol
ATP Production
OCR (pmol/min/µg)
0
20
40
60
80
Vehicle
Aramchol

FigS6

Supplementary Fig 6. H iHLCs, treatment with Aramchol did not alter LD formation or improve mitochondrial function A. Representative confocal image (40X objective) of H iHLCs treated with vehicle or Aramchol and stained with Neutral lipid stain (green) and nuclear stain (blue). B. Quantitative measurements of parameters of basal respiration, maximal respiration, spare respiratory capacity and ATP production in AC iHLCs compared to control iHLCs.

A

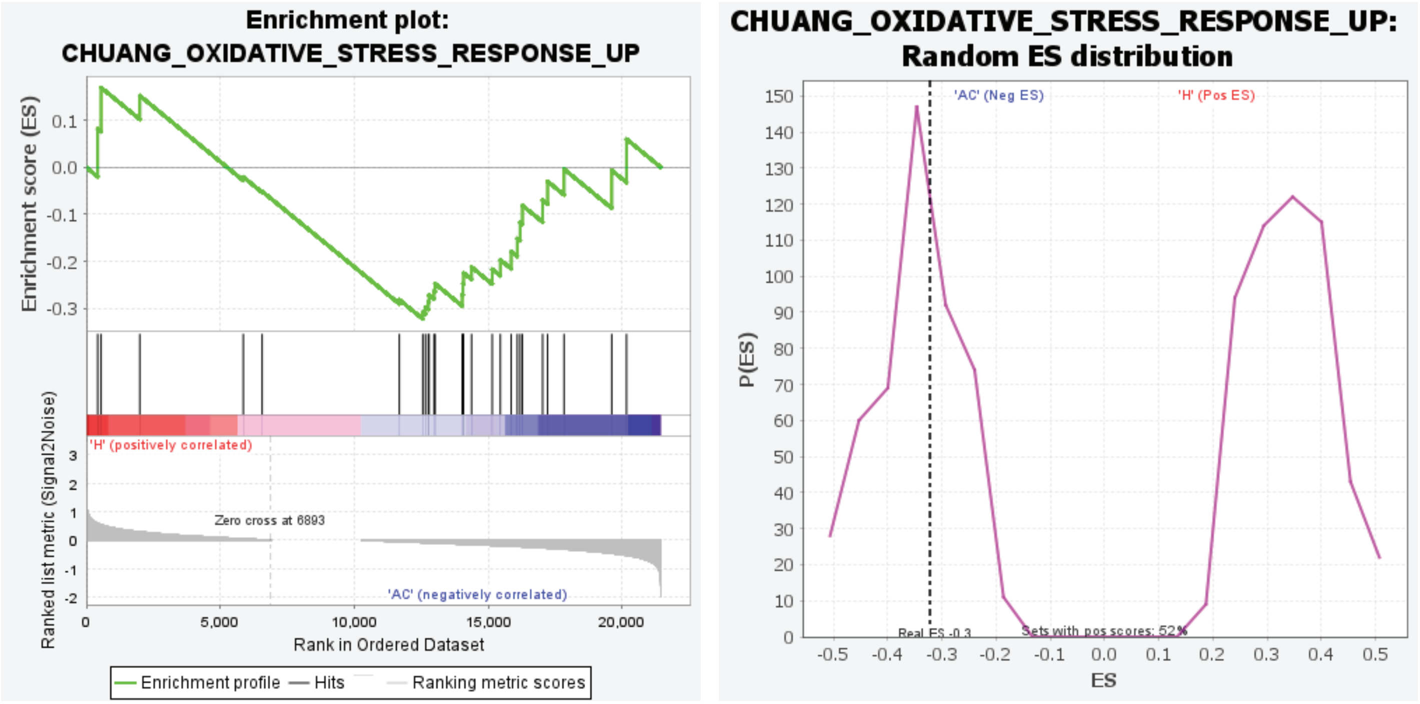

B

Table: Gene sets enriched in phenotype AC

GS	SIZE	ES	NES	FDR q-val	LEADING EDGE
REGULATION_OF_OXIDATIVE_STRESS_INDUCED_CELL_DEATH	46	-0.37	-1.37	1	tags=46%, list=34%, signal=69%
REGULATION_OF_OXIDATIVE_STRESS_INDUCED_INTRINSIC_APOPTOTIC_SIGNALING_PATHWAY	20	-0.43	-1.32	1	tags=55%, list=34%, signal=83%
CELL_DEATH_IN_RESPONSE_TO_OXIDATIVE_STRESS	90	-0.29	-1.26	0.973	tags=39%, list=34%, signal=59%
NEGATIVE_REGULATION_OF_RESPONSE_TO_OXIDATIVE_STRESS	19	-0.42	-1.24	0.968	tags=32%, list=20%, signal=40%
REGULATION_OF_OXIDATIVE_STRESS_INDUCED_CELL_DEATH	68	-0.28	-1.19	0.94	tags=43%, list=37%, signal=68%
RESPONSE_TO_OXIDATIVE_STRESS	421	-0.21	-1.08	0.859	tags=33%, list=34%, signal=48%
REGULATION_OF_RESPONSE_TO_OXIDATIVE_STRESS	89	-0.23	-1.05	0.865	tags=42%, list=37%, signal=66%
INTRINSIC_APOPTOTIC_SIGNALING_PATHWAY_IN_RESPONSE_TO_OXIDATIVE_STRESS	45	-0.24	-0.95	0.867	tags=42%, list=34%, signal=64%

Supplementary Fig 7. Transcriptomic enrichment of DEGs gene highlighting the role of oxidative stress associate pathways A. Enrichment plot from GSEA analyses using Chuang oxidative stress response geneset and its random ES distribution. B.A table of oxidative stress associated pahways from GSEA analyses implicated by transcriptome analyses of AC iHLCs.

FigS7

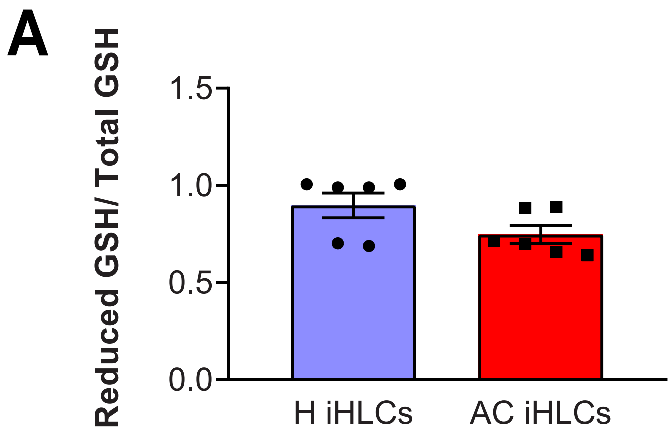

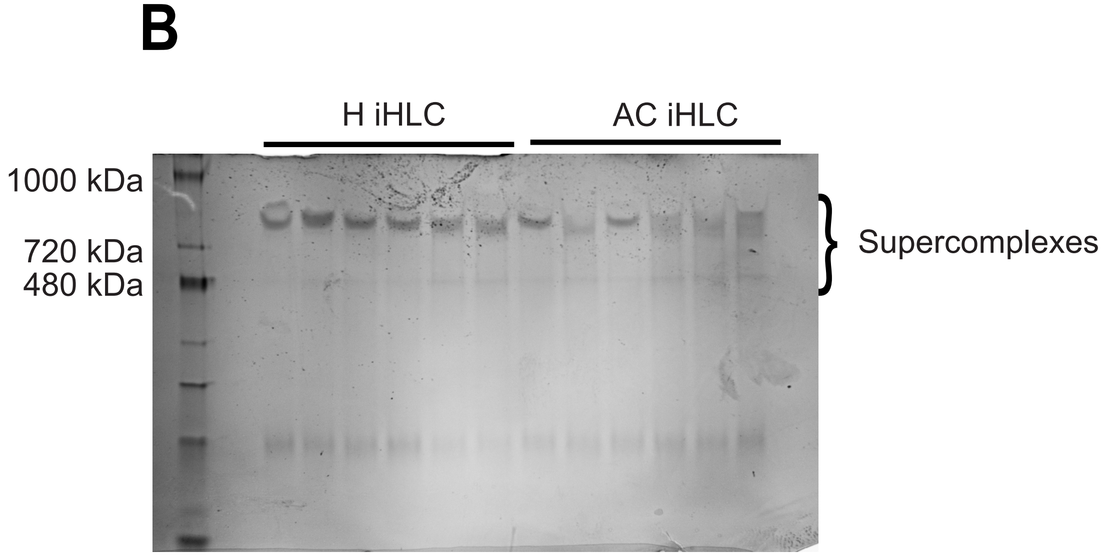

Supplementary Fig 8. Mitochondrial supercomplex ssembly and antioxidant levels of isolated mitochondria isolated from of H and AC iHLCs A. Mitochondrial antioxidant capacity evaluated using the ratio of reduced glutathione to total glutathione . A nonsignificant trend for reduced capacity was observed in AC iHLCs . B. Isolated mitochondrial membrane fractions were run in native gel and a mobility shift was observed between healthy and AC iHLCs group indicating a possible difference in assembly of mitochondrial super complexes. B.

FigS8

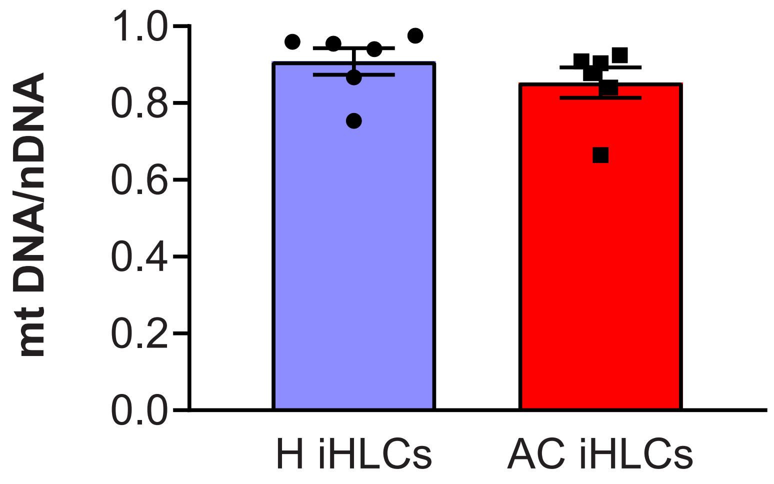

Supplementary Fig 9. The mitochondrial DNA to nuclear DNA ratio in iHLCs. There was no statistical difference between H iHLCs and AC iHLCs in mtDNA, n = 6/groups.

FigS9
